# Hormone Activity of Hydroxylated Polybrominated Diphenyl Ethers on Human Thyroid Receptor-β: *In Vitro* and *In Silico* Investigations

**DOI:** 10.1289/ehp.0901457

**Published:** 2009-12-17

**Authors:** Fei Li, Qing Xie, Xuehua Li, Na Li, Ping Chi, Jingwen Chen, Zijian Wang, Ce Hao

**Affiliations:** 1 Key Laboratory of Industrial Ecology and Environmental Engineering, School of Environmental Science and Technology, Dalian University of Technology, Dalian, China; 2 State Key Laboratory of Environmental Aquatic Chemistry, Research Center for Eco-environmental Sciences, Chinese Academy of Sciences, Beijing, China; 3 Carbon Research Laboratory, Center for Nano Materials and Science, School of Chemical Engineering, State Key Laboratory of Fine Chemicals, Dalian University of Technology, Dalian, China

**Keywords:** application domain, density functional theory, docking, HO-PBDEs, hydroxylated polybrominated diphenyl ethers, PBDEs, quantitative structure-activity relationship, thyroid hormone receptor

## Abstract

**Background:**

Hydroxylated polybrominated diphenyl ethers (HO-PBDEs) may disrupt thyroid hormone status because of their structural similarity to thyroid hormone. However, the molecular mechanisms of interactions with thyroid hormone receptors (TRs) are not fully understood.

**Objectives:**

We investigated the interactions between HO-PBDEs and TRβ to identify critical structural features and physicochemical properties of HO-PBDEs related to their hormone activity, and to develop quantitative structure–activity relationship (QSAR) models for the thyroid hormone activity of HO-PBDEs.

**Methods:**

We used the recombinant two-hybrid yeast assay to determine the hormone activities to TRβ and molecular docking to model the ligand–receptor interaction in the binding site. Based on the mechanism of action, molecular structural descriptors were computed, selected, and employed to characterize the interactions, and finally a QSAR model was constructed. The applicability domain (AD) of the model was assessed by Williams plot.

**Results:**

The 18 HO-PBDEs tested exhibited significantly higher thyroid hormone activities than did PBDEs (*p* < 0.05). Hydrogen bonding was the characteristic interaction between HO-PBDE molecules and TRβ, and aromaticity had a negative effect on the thyroid hormone activity of HO-PBDEs. The developed QSAR model had good robustness, predictive ability, and mechanism interpretability.

**Conclusions:**

Hydrogen bonding and electrostatic interactions between HO-PBDEs and TRβ are important factors governing thyroid hormone activities. The HO-PBDEs with higher ability to accept electrons tend to have weak hydrogen bonding with TRβ and lower thyroid hormone activities.

Polybrominated diphenyl ethers (PBDEs) have become ubiquitous environmental pollutants because of their historical and widespread use as flame retardants, and they have received great attention from ecological health and environmental perspectives (Athanasiadou et al. 2008; He et al. 2008; Lagalante et al. 2009). Most recently, hydroxylated PBDEs (HO-PBDEs) have caused increasing concern because of reports of their natural production and metabolism (Hakk and Letcher 2003; Malmberg et al. 2005; Malmvarn et al. 2005; Mercado-Feliciano and Bigsby 2008; Wan et al. 2009). HO-PBDEs have been detected in the blood of fish, birds, and mammalian species and even in the abiotic environment (Athanasiadou et al. 2008; Cantón et al. 2008; Lacorte and Ikonomou 2009). Recent studies (Dingemans et al. 2008; Qiu et al. 2009) suggest that some of the toxic effects of PBDEs might be due to their HO metabolites.

*In vitro* tests have shown that certain PBDEs can affect thyroid hormone homeostasis by acting as potent competitors of thyroid hormones for binding to human transthyretin (TTR) and thyroid hormone receptors (TRs) (Cantón et al. 2006; Darnerud et al. 2007; Zhou et al. 2002). For instance, levels of serum thyroxine (T_4_) were significantly decreased when rats were exposed to PBDEs (Zhou et al. 2002). The effects of PBDEs on T_4_ levels may require metabolic activation because HO-PBDEs, but not the PBDE congeners themselves, behave as ligands for human TTR *in vitro* (Meerts et al. 2000; Qiu et al. 2007). Kitamura et al. (2008) also reported that 4-OH-BDE-90 and 3-OH-BDE-47 markedly inhibited the binding of triiodothyronine (T_3_) to TRα and acted as thyroid hormone-like agents. Recently, Kojima et al. (2009) reported that 4-HO-BDE-90 significantly inhibited TRα- and TRβ-mediated transcriptional activity induced by T_3_. Consequently, HO-PBDEs have attracted great attention (Routti et al. 2009). However, there is lack of systematic investigation into the mechanisms by which HO-PBDEs interfere with hormonal actions (Zhao et al. 2008).

There are mainly two subtypes of TRs, TRα and TRβ, expressed from two different genes. TRα mediates the effects of thyroid hormones on the heart, in particular on heart rate and rhythm, whereas most actions of the hormones on the liver and other tissues are mediated more through TRβ (Forrest and Vennstrom 2000). The initial step for chemical mode of action is binding to an intracellular receptor (Kavlock et al. 1996). Given the large number of compounds that may bind to the receptors, there is increasing interest in developing computational methods (*in silico*) to predict affinity of compounds with the receptors, including quantitative structure–activity relationships (QSARs) (Du et al. 2008; Valadares et al. 2007). Furthermore, molecular docking and virtual screening have become an integral part of many modern structure-based computational simulations of chemicals (Martinez et al. 2008). Docking methodologies use the knowledge of three-dimensional structure of a receptor in an attempt to optimize the bound ligand or a series of molecules into the active site. Combinational use of docking with QSAR can provide more information on the interaction between the ligand and the receptor (Sippl 2002; Soderholm et al. 2005).

In this study, we applied an integrated *in vitro* and *in silico* approach to evaluate the thyroid hormone–disrupting potency of HO-PBDEs and to identify critical structural elements and physicochemical properties of HO-PBDEs related to their hormone activity. The hormone activities of 18 HO-PBDEs to human TRβ were determined using the recombinant two-hybrid yeast assay. Molecular docking was performed to find the significant ligand–receptor interactions in the binding site of TRβ, which provided a better understanding of interactions between the HO-PBDEs and TRβ. Molecular structural descriptors were computed, selected, and employed to characterize the interactions, and finally we constructed QSAR models. We also assessed the applicability domain (AD) of the QSAR model.

## Materials and Methods

### Chemicals

We selected 18 HO-PBDEs for the present study [see Supplemental Material, Figure S1 (doi:10.1289/ehp.0901457)]; most have been detected in environmental/biological samples (Malmberg et al. 2005; Qiu et al. 2007). The HO-PBDEs congeners (> 97% purity) were purchased from AccuStandard (New Haven, CT, USA). 3,3′,5-Triiodothyronine (T_3_; 95% purity), dimethyl sulfoxide (DMSO; GC, 99.5% purity), *o*-nitrophenyl β-d-galactopyranoside (*o*-NPG; ≥ 98% purity), sodium dodecyl sulfate (99% purity), leucine (99% purity), tryptophan (99% purity), yeast-based nitrogen (99% purity), and β-mercaptoethanol (99% purity) were purchased from Sigma Chemical Company (St. Louis, MO, USA). Stock solutions of HO-PBDEs were prepared in DMSO.

### Recombinant two-hybrid yeast assay and statistical analysis

The recombinant two-hybrid yeast system employed a yeast cell transformed with the human TRβ plasmid, coactive plasmid, and the reporter gene expressing β-galactosidase (Li et al. 2008). We examined the specificity of the yeast two-hybrid assay for TRβ ligand using DMSO (control), T_3_, and other steroid hormones. T_3_ induced β-galactosidase activity, whereas 17β-estradiol, dihydrotestosterone, and progesterone did not. Thus, the recombinant two-hybrid yeast assay was highly specific for TRβ ligand without cross-talk to other receptor agonists.

We performed the recombinant two-hybrid yeast assay as described previously by Li et al. (2008). Briefly, yeast transformants were grown overnight at 30°C, with vigorous orbital shaking (130 rpm). For the assay, exponentially growing overnight cultures were diluted with synthetic dextrose/leucine/tryptamine medium to an optical density at 600 nm (OD_600_) of 0.75. All the samples were determined at least in triplicate. Each triplicate included a positive control (T_3_) and a negative control (DMSO). Each tested chemical was serially diluted in DMSO for a total of 7–11 concentrations. Serial dilutions (5-μL steps) were combined with 995 μL medium containing 5 × 10^3^ yeast cells/mL, resulting in a test culture in which the volume of DMSO did not exceed 0.5% of the total volume. For each test culture, 200 μL was transferred into a well of the 96-well plate and incubated at 30°C with vigorous orbital shaking (800 rpm) on a titer plate shaker (TITRAMAX 1000; Heidolph Instruments GmbH, Hamburg, Germany) for 2 hr, and the cell density of the culture was measured at OD_600_ (GENios A-5002; Tecan Austria GmbH, Salzburg, Austria). Then, 50 μL test culture was transferred to a new 96-well plate, and after addition of 120 μL Z-buffer (21.51 g/L Na_2_HPO_4_·12H_2_O; 6.22 g/L NaH_2_PO_4_·2H_2_O; 0.75 g/L KCl; 0.25 g/L MgSO_4_·7H_2_O) and 20 μL chloroform, the cultures were carefully mixed and preincubated for 10 min at 30°C and 13,000 rpm. The enzyme reaction was started by adding 40 μL *o*-NPG (13.3 mM, dissolved in yeast-based buffer). The assay culture was further incubated at 30°C for 1 hr. Finally, the reactions were terminated by the addition of 100 μL sodium carbonate (1 M). The resulting absorption was measured at 420 nm. The β-galactosidase activity (*U*) was calculated according to the following equation:


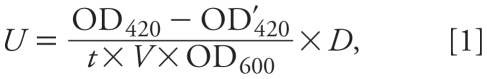


where *U* is the activity of β-galactosidase, *t* is the incubation duration of the enzyme reaction, *V* is the volume of the test culture, *D* is the diluting factor (6.6), OD_600_ is the cell density measured at 600 nm, and OD_420_ and OD′_420_ are the cell density of the enzymic reaction supernatant and the blank, respectively, measured at 420 nm.

The dose–response curves for *U* of the tested compounds were fitted by iterative four-parameter curve fit method using SigmaPlot, version 10.0 (Systat Software Inc., Chicago, IL, USA). The concentration inducing 20% of the maximum effect (REC_20_) value was calculated from the fitted dose–response curves. We evaluated the statistical significance of differences by analysis of variance (we considered *p* < 0.05 significant).

### Molecular docking

We adapted the CDOCKER algorithm to find the binding mode for HO-PBDEs to TRβ. CDOCKER is an implementation of a CHARMM (Chemistry at HARvard Macromolecular Mechanics)-based docking tool that has been shown to be viable (Wu et al. 2003). It has been incorporated into Discovery Studio 2.1 (Accelrys Software Inc., San Diego, CA) through the Dock Ligands (CDOCKER) protocol. We extracted the crystal structure of TRβ (Thyroid receptor beta1 in complex with a beta-selective ligand; PDB ID 1NAX) from the RCSB Protein Data Bank [RCSB (Research Collaboratory for Structural Bioinformatics) PDB; http://www.rcsb.org/pdb]. In CDOCKER, random ligand conformations were generated from the initial ligand structure through high-temperature molecular dynamics followed by random rotations. Then, the random conformations were refined by grid-based simulated annealing, which makes the results more accurate. The CDOCKER interaction energy between the ligand and TRβ (*E*_binding_) was then computed. The docking analysis provided insights into the interactions between the ligands and the receptor, which facilitated the selection of appropriate molecular parameters to characterize the interactions in the QSAR studies.

### Molecular structural descriptor generation and QSAR development

We hypothesized that the thyroid hormone activities of HO-PBDEs were dependent on *a*) the partition of the compounds between water and the biophase, and *b*) the interaction between the ligands and the receptor TRβ. Thus, 12 theoretical parameters were computed and selected to characterize the processes: the logarithm of octanol/water partition coefficient (log*K*_ow_), average molecular polarizability (α), molecular volume (*V*), dipole moment (μ), energy of the highest occupied molecular orbital (*E*_HOMO_), energy of the lowest unoccupied molecular orbital (*E*_LUMO_), formal charge on hydroxyl hydrogen atoms (*q*_O__H_), formal charge on hydroxyl oxygen atoms (*q*_O__H_), formal charge on ether oxygen atoms (*q*_O_), electrophilicity index (ω), harmonic oscillator model of aromaticity index (*I*_A_), and the number of bromine atoms (*n*_Br_) [see also Supplemental Material, Table S1 (doi:10.1289/ehp.0901457)]. We purposely selected log*K*_ow_ to describe the partition process. The parameters *V*, *n*_Br_, α, and μ also partly describe partition because many of these parameters correlate with log*K*_ow_ (Nguyen et al. 2005). The parameters *E*_HOMO_, *E*_LUMO_, *q*_O__H_, *q*_O__H_, *q*_O_, ω, and *I*_A_ were purposely selected to describe the intermolecular electrostatic interactions between the ligands and TRβ. The quantum chemical parameters *E*_HOMO_, *E*_LUMO_, *q*_O__H_, *q*_O__H_, and *q*_O_ proved successful in many QSAR studies for characterizing intermolecular electrostatic interactions (Colosi et al. 2006). ω measures the ability of a compound to soak up electrons. The relative binding affinity of some estrogen derivatives correlated strongly with ω (Chattaraj 2006). The aromaticity of compounds (*I*_A_) may influence their noncovalent interactions with the receptor, and *I*_A_ has been used to characterize halogenated biphenyls (Alonso et al. 2008).

We computed log*K*_ow_ values using the EPI Suite, version 4.0 (U.S. Environmental Protection Agency 2009). *V* (defined as the volume inside a contour of 0.001 electrons/bohr^3^ density) and the quantum chemical parameters were computed by the Gaussian 03 programs (Frisch et al. 2004). Initial geometries of HO-PBDEs were preoptimized by semiempirical PM3 Hamiltonian and then optimized by density functional theory at the B3LYP/6-311+G(d,p) level. Frequency analysis was performed on the optimized geometries to ensure the systems had no imaginary vibration frequencies. *I*_A_ was calculated by DRAGON software (Todeschini and Consonni 2000).

The 18 HO-PBDEs were randomly divided into a training set (80%) and a validation set (20%), as listed in Table 1. Partial least squares (PLS) regression was performed in developing the model because PLS can analyze data with strongly collinear, noisy, and numerous predictor variables (Wold et al. 2001). We used Simca-S (version 6.0; Umetri AB, Umea, Sweden) for the PLS analysis. Simca-S uses leave-many-out cross-validation to determine the number of PLS components (*A*). Cross-validation simulates how well a model predicts new data and gives a statistical fraction of the total variation of the dependent variables that can be predicted by all the extracted components (*Q*^2^_CUM_) for the final model. The PLS analysis was performed repeatedly to eliminate redundant molecular structural parameters, as done in our previous studies (Chen et al. 2004). Model predictability was evaluated by external validation, which was characterized by the determination coefficient (*R*^2^), root mean square error (RMSE), and external explained variance (*Q*^2^_EXT_), which are defined as follows (Schüürmann et al. 2008):


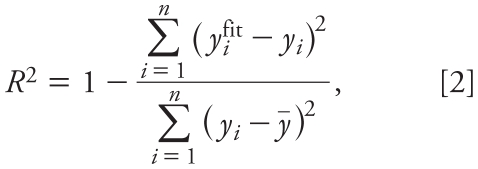



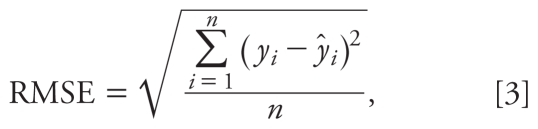



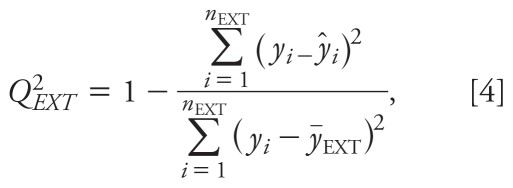


where *y_i_*^fit^ is the fitted − logREC_20_ value of the *i*th compound; *ȳ* is the average response value in the training set; *y_i_* and *ŷ_i_* are the observed and predicted values for the *i*th compound, respectively; *ȳ_EXT_* is the average response value of the validation set; *n* is the number of compounds in the training set; and *n*_EXT_ is the number of compounds in the validation set.

We assessed the AD of the developed QSAR model using the Williams plot, that is, the plot of standardized residuals (σ) versus leverage (hat diagonal) values (*h_i_*) (Eriksson et al. 2003). We calculated σ as follows:


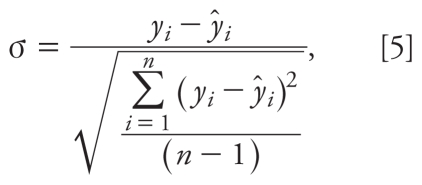


where *y*_i_ and *ŷ_i_* are the observed and predicted values for the *i*th compound, respectively, and *n* is the number of compounds in the training set.

The *h_i_* value of a chemical in the original variable space and the warning leverage value (h*) are defined as follows:


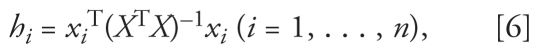



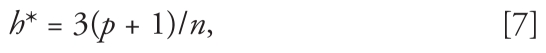


where *x_i_* is the descriptor vector of the considered compound, *X* is the model matrix derived from the training set descriptor values, and *p* is the number of predictor variables.

## Results and Discussion

### Thyroid hormone activity determined by recombined yeast

Based on a plot of *U* versus log T_3_ concentrations, the maximal induction of T_3_ was achieved at 1.0 × 10^−6^ M [see Supplemental Material, Figure S2 (doi:10.1289/ehp.0901457)]. From the dose–response curve, the median effective concentration value of T_3_ was 1.4 × 10^−7^ M, which was similar to that reported by Li et al. (2008). The 18 tested HO-PBDEs induced β-galactosidase activity in a concentration-dependent manner in the concentration range from 10^−11^ to 10^−6^ M (see Supplemental Material, Figure S3). Supplemental Material, Table S2 lists the determined REC_20_ values for the 18 HO-PBDEs.

With 2 × 10^−7^ M of the tested compounds, the PBDEs showed no significant β-galactosidase activity compared with DMSO, and HO-PBDEs exhibited significant activity [*p* < 0.05; see Supplemental Material, Figure S4 (doi:10.1289/ehp.0901457)]. Previous studies (Hamers et al. 2008; Meerts et al. 2000) indicated that HO metabolites of PBDEs could compete with T_4_ for binding to TTR and exert thyroid hormone activity. Kitamura et al. (2008) reported that 4-OH-BDE-90 and 3-OH-BDE-47 markedly inhibited the binding of T_3_ to TRα and acted as thyroid hormone-like agents. Kojima et al. (2009) reported that 4-HO-BDE-90 significantly inhibited TRα- and TRβ-mediated transcriptional activity induced by T_3_. Thus, with respect to the observed thyroid hormone activity of HO-PBDEs, our results are consistent with previous findings.

### Docking analysis

**Figure 1:**
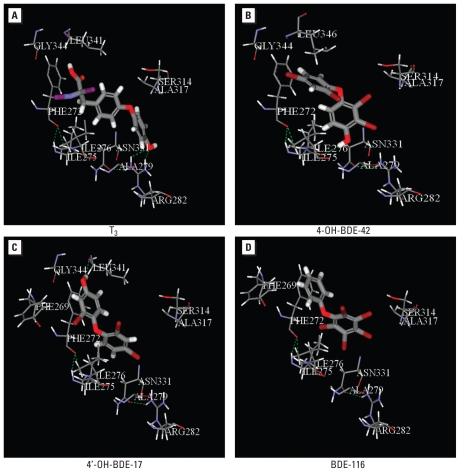
Docking views of T_3_ (*A*), 4-OH-BDE-42 (*B*), 4′-OH-BDE-17 (*C*), and BDE-116 (*D*) at the TRβ binding site. Green dashed lines indicate hydrogen bonds between HO-PBDEs and amino acid residues, gray is carbon, red is oxygen, blue is nitrogen, purple is iodine, and white is hydrogen.

Figure 1 shows the docking view of T_3_ and representative HO-PBDEs and PBDEs (4-OH-BDE-42, 4′-OH-BDE-17, and BDE-116) in the binding site of TRβ. At the deep end of the pocket, Arg282 and Ile275 serve as anchoring points for the ligands. The ligands also interact with the second polar region within the binding pocket, Leu341. We observed hydrogen bonding to be a characteristic interaction. As shown in Figure 1B and C, there are mainly two types of hydrogen bonds: those formed between the hydroxyl oxygen of HO-PBDEs and the hydrogen of Arg282 and Ile276, and those between the hydroxyl hydrogen of HO-PBDEs and the carbonyl oxygen of Leu341. However, for BDE-116, we could find no hydrogen bonds with the amino acid residues in TRβ. We also observed π–π interactions between the phenyl of HO-PBDEs and Phe272, Phe442, and Phe455.

**Figure 2:**
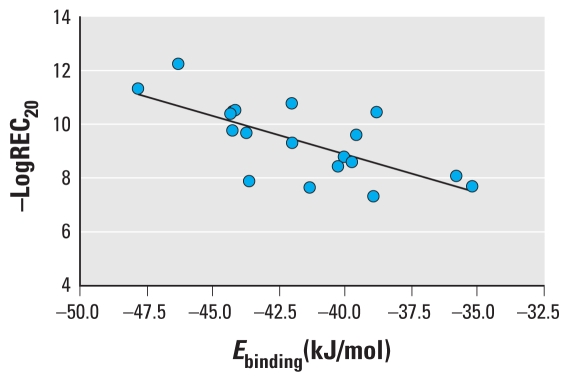
Plot of observed − logREC_20_ versus the *E*_binding_. *R* = 0.685; *p* < 0.02.

The ligand–receptor binding energy (*E*_binding_) of the 18 HO-PBDEs is listed in Table 1. As shown in Figure 2, we obtained a simple linear free energy relationship between –logREC_20_ and *E*_binding_, further proving that binding to TRβ is the key step for the HO-PBDEs to exert their thyroid hormone activity. However *E*_binding_ itself was not a good predictor for − logREC_20_, as indicated by the big prediction residuals for some HO-PBDEs (Figure 2). This was expected because *E*_binding_ values calculated by CDOCKER differed from the real binding energy because the environmental factors (e.g., solvents, pH, and ions) were not considered in the modeling, and the ability of β-galactosidase expression for different HO-PBDEs may differ. Thus, it is necessary to develop multiparameter QSAR models for − logREC_20_ prediction.

### Development, validation, and AD of the QSAR

PLS analysis with − logREC_20_ as the dependent variable and the molecular structural parameters as predictor variables resulted in the following optimal QSAR model:


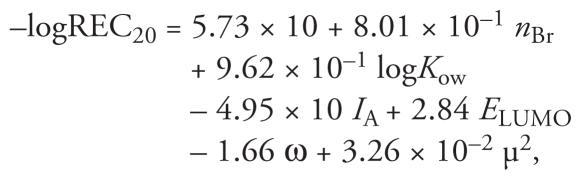


where *n* = 14, *A* = 3, *R*^2^ = 0.913, *Q*^2^_CUM_ = 0.873, RMSE (training set) = 0.418, *n*_EXT_ = 4, *Q*^2^_EXT_ = 0.500, and RMSE (validation set) = 0.731 (*p* < 0.0001).

**Figure 3:**
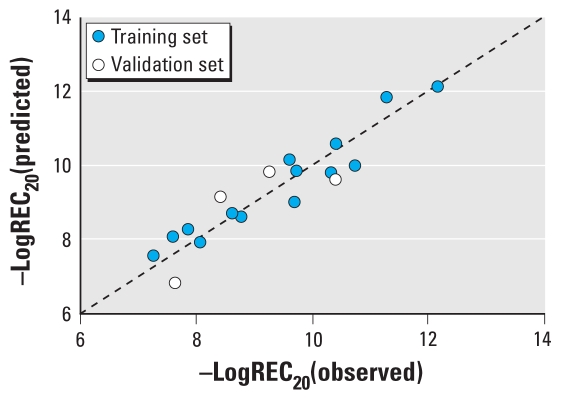
Plot of predicted versus observed − logREC_20_ values for both the training set (*R*^2^*_Y_* = 0.913; RMSE = 0.418) and the validation set (*Q*^2^_EXT_ = 0.500; RMSE = 0.731).

*Q*^2^_CUM_ of the QSAR was as high as 0.873, implying good robustness of the model. The differences between *R*^2^ and *Q*^2^_CUM_ did not exceed 0.3, indicating no overfitting in the model (Golbraikh and Tropsha 2002). Figure 3 shows that the predicted − logREC_20_ values are consistent with the observed values for both the validation and training sets. The model has good predictive abilities, as indicated by *Q*^2^_EXT_ = 0.500 and RMSE = 0.731.

**Figure 4:**
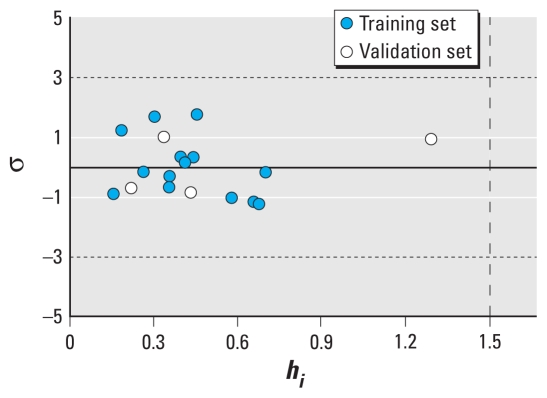
Williams plot showing AD of the developed QSAR model (see also Equations 5 and 6). The vertical dashed line indicates that *h_i_* = h* = 1.500.

The AD is shown by the Williams plot (Figure 4); *h_i_* values of all the compounds in the training and validation sets were lower than the warning value (h* = 1.500). Thus, none of the compounds are particularly influential in the model space, and the training set has great representativeness. The standardized residuals of all the compounds in the training and validation sets are < 3, so there are no outliers in the developed QSAR model. Considering the mechanism of action, we can infer that the developed QSAR model can be used to predict thyroid hormone activity on TRβ of other HO-PBDEs similar to those used in the present study. To discriminate between active and inactive compounds (e.g., PBDEs and HO-PBDEs), discriminant models should be developed in further studies.

### Mechanistic implication of the developed model

The developed PLS model extracted on three PLS components loaded primarily on six predictor variables. Values of the variable importance in the projection (VIP) and PLS weights (W*) are listed in Table 2. From the W* values, one can see how the predictor variables and the response variable combine in the projections (PLS components) and how they relate to each other.

The first PLS component (W*c[1]) is loaded primarily on three descriptors, *n*_Br_, log*K*_ow_, and *I*_A_ (Table 2). These three descriptors remarkably govern − logREC_20_ values, as indicated by their large VIP values among the predictor variables. The coefficients in the QSAR model indicate that − logREC_20_ values of HO-PBDEs increase with *n*_Br_ and log*K*_ow_ values but decrease with increasing *I*_A_ values. The observation is reasonable because log*K*_ow_ correlates with *n*_Br_ positively (*r* = 0.999, *p* < 0.001) and because HO-PBDEs with large log*K*_ow_ and *n*_Br_ values tend to partition into the biophase (yeast cells). The harmonic oscillator model of aromaticity index (*I*_A_) may characterize the noncovalent interactions with TRβ. The deviation from planarity of an aromatic ring is a structural measurement of aromaticity: The higher the planarity, the higher the π electron delocalization and the higher the aromaticity (Alonso et al. 2008). Even for a single ring of polychlorinated biphenyl, aromaticity varied with the number of chlorine atoms (Alonso et al. 2008). Thus, the involvement of *I*_A_ in the QSAR model may imply the effects of planarity (or nonplanarity) of HO-PBDE molecules on thyroid hormone activity. According to the QSAR model, *I*_A_ plays a negative effect on thyroid hormone activity (*n* = 18; *r* = 0.66; *p* < 0.005). *E*_binding_ also correlates positively with *I*_A_ values (*n* = 18; *r* = 0.54; *p* < 0.05).

The second PLS component (W*c[2]) is loaded primarily on *I*_A_, *E*_LUMO_, ω, and μ^2^, and the third PLS component (W*c[3]) is loaded primarily on *n*_Br_, log*K*_ow_, *E*_LUMO_, and ω. *E*_LUMO_ itself has a negative value and measures the ability of a molecule to accept electrons. The docking analysis showed that hydrogen bonds were the characteristic interactions between the hydroxyl oxygen of HO-PBDEs and the hydrogen of Arg282 and Ile276. Because HO-PBDE molecules with lower *E*_LUMO_ values tend to accept electrons easily, accept protons with difficulty, and accordingly form the hydrogen bonds with difficulty, the developed PLS model shows that − logREC_20_ increases with *E*_LUMO_ values. Likewise, because ω measures the ability of a molecule to soak up electrons (Chattaraj 2006), in the developed PLS model − logREC_20_ increases with decreasing ω values. Finally, μ measures the dipole–dipole and dipole-induced interactions between interacting molecules (Hurth et al. 2004), so HO-PBDEs with large μ values may exhibit strong dipole–dipole interactions with the receptor (TRβ), leading to large − logREC_20_ values.

## Conclusion

We determined thyroid hormone activities of selected HO-PBDEs by the recombinant two-hybrid yeast assay. Docking analysis indicated that hydrogen bonding and electrostatic interactions are the key steps for HO-PBDEs to exert thyroid hormone activities. We developed a QSAR to characterize the interactions and model the thyroid hormone activities. The HO-PBDEs with higher ability to accept electrons (as indicated by *E*_LUMO_ and ω) tend to have weak hydrogen bonding with the receptor and lower thyroid hormone activities. The developed QSAR model has good robustness, predictive ability, and mechanism interpretability.

## Figures and Tables

**Table 1:** − LogREC_20_ and *E*_binding_ values for selected compounds.

	− LogREC_20_			
Compound	Observed	Predicted	Residual	*E*_binding_ (kJ/mol)
3′-OH-BDE-7^a^	7.64	7.03	0.61	−35.3
4′-OH-BDE-17	8.66	9.05	−0.39	−39.8
3′-OH-BDE-28	7.28	7.75	−0.47	−38.9
2′-OH-BDE-28	8.07	7.95	0.12	−35.8
4-OH-BDE-42	9.72	8.90	0.82	−43.7
4′-OH-BDE-49	7.87	8.31	−0.44	−43.6
3-OH-BDE-47	8.77	8.91	−0.14	−40.0
5-OH-BDE-47^a^	8.44	9.18	−0.74	−40.2
6-OH-BDE-47^a^	10.43	9.85	0.58	−38.8
4-OH-BDE-90	7.63	7.96	−0.33	−41.3
6-OH-BDE-85	9.77	9.79	−0.02	−44.3
6-OH-BDE-87^a^	9.29	9.65	−0.36	−42.0
6-OH-BDE-82	10.44	10.55	−0.11	−44.2
6′-OH-BDE-99	9.62	9.93	−0.31	−39.6
5′-OH-BDE-99	10.34	9.73	0.61	−44.4
6-OH-BDE-157	12.20	11.97	0.23	−46.3
6-OH-BDE-140	11.31	11.80	−0.49	−47.8
3′-OH-BDE-154	10.76	9.91	0.85	−42.0
BDE-30	< 6.70			−37.0
BDE-116	< 6.70			−34.4

a Compound selected to form the external validation set.

**Table 2:** VIP values and PLS weights for the optimal PLS model.

Variable	VIP	W*c[1]	W*c[2]	W*c[3]
*n*_Br_	1.093	0.487	0.194	0.585
Log*K*_ow_	1.091	0.487	0.191	0.581
*I*_A_	1.022	−0.420	−0.589	−0.246
*E*_LUMO_	0.980	−0.387	0.469	0.615
ω	0.979	0.392	−0.451	−0.592
μ^2^	0.806	0.215	0.428	−0.345
